# Factors Affecting the Survival of Patients with Oligometastatic Non-Small-Cell Lung Cancer: A Meta-Analysis

**DOI:** 10.1155/2019/2153170

**Published:** 2019-05-19

**Authors:** Yu Shi, Jianxin Yang, Ninghua Yao, Minghai Shao, Wenxiu Ding, Wanrong Jiang, Xinchen Sun

**Affiliations:** ^1^Department of Radiotherapy, The First Affiliated Hospital of Nanjing Medical University, Nanjing, Jiangsu Province, China; ^2^Department of Radiotherapy, Affiliated Hospital of Nantong University, Nantong, Jiangsu, China; ^3^Department of General Surgery, Qidong People's Hospital, Qidong Liver Cancer Institute, Nantong, Jiangsu, China; ^4^Department of Radiotherapy, Taizhou Hospital of Zhejiang Province, Taizhou, Zhejiang Province, China; ^5^Department of Radiotherapy, Taixing People's Hospital, Taizhou, Jiangsu Province, China; ^6^Department of Radiotherapy, People's Liberation Army (PLA) 81 Hospital, Nanjing, Jiangsu Province, China

## Abstract

**Background:**

The aim was to investigate the potential factors related with overall survival of oligometastatic non-small-cell lung cancer (NSCLC) patients.

**Methods:**

A literature search was conducted in databases including PubMed, Embase, and Cochrane library up to March 2017. The hazard radio (HR) as well as the corresponding 95% confidence interval (CI) were calculated, and all the statistics analysis was performed by the R 3.12. Heterogeneity was analyzed using I-squared and Cochran Q tests. Furthermore, sensitivity analysis was performed to evaluate the stability of results.

**Results:**

In total, 6 articles were included in the meta-analysis. Nodal status was significantly correlated with the overall survival rate of NSCLC oligometastatic patients (HR: 1.69, 95% CI: 1.23–2.32, *Z*=3.20, *P*=0.001). No significant relationship was found between overall survival rate of NSCLC oligometastatic patients and the indicators including sex, stage, smoker, age, and histology. Notably, sensitivity analysis on data evaluating relationship between patients survival and the stage and histology showed that results were reversed after removing one of the studies.

**Conclusions:**

Nodal status might be associated with the overall survival of oligometastatic NSCLC patients.

## 1. Background

Lung cancer is one of the leading causes of cancer-related deaths, and 80% among them are non-small-cell lung cancer (NSCLC) worldwide [1, 2]. Due to limited diagnostic technology, most NSCLC patients are diagnosed with advanced cancer [1, 3], and almost half among them have distant metastases (such as brain, adrenal glands, bone, or liver) [3, 4]. Although treatment technology develops continually, the survival of NSCLC patients is still poor due to the metastases [5, 6].

Oligometastasis is a notion that cancer patients develop 1–5 metastatic or recurrent lesions after treatment, which will affect the survival of patients [7]. Among them, patients with 1-2 metastases and recurrences show better prognosis than those with 3–5 metastases and recurrences [8]. Moreover, it has been reported that local therapy such as radiotherapy and surgery can effectively improve the survival of patients with postoperative oligometastases [9].

A recent meta-analysis put forward several prognostic factors associated with oligometastatic NSCLC treatment efficacy based on data of 757 individual patients [10]. The association between these factors (demographics and tumor status) and the survival of NSCLC patients with postoperative oligometastases has been investigated in previous studies. Several factors associated with overall survival evaluation have been reported as opposed to metachronous appearance of oligometastases [11–14]. However, the results of these studies are controversial. The study by Parikh et al. demonstrates that nodal involvement, pathology, and patient performance status may influence survival of oligometastatic NSCLC patients [15]. However, other studies do not support the above relationship [9, 16]. For example, the study by Griffioen et al. also does not find any association between the overall survival after oligometastatic NSCLC treatment and histology, age, smoke, and stage [17]. Therefore, it is not clear whether patient outcomes would have differed significantly if these oligometastatic NSCLC patients had various backgrounds.

Although these factors have been studied in several previous studies, the sample size and study content are limited in one clinical study. Therefore, in this study, we performed a meta-analysis pooling data of previously published clinical studies to systematically analyze the data and analyze the risk factors associated with the overall survival of NSCLC patients.

## 2. Materials and Methods

### 2.1. Literature Search Strategy

Databases including PubMed (https://www.ncbi.nlm.nih.gov/pubmed), Embase (https://www.embase.com/), and Cochrane library (http://www.cochranelibrary.com/) were used for searching the English literature correlated with oligometastatic NSCLC. The search period was up to March 12, 2017. The keywords used for literature searching in Embase and Cochrane library mainly included (“Oligometastatic” or “oligometastasis”) AND (“NSCLC” or “nonsmall cell lung cancer”). The searching words in PubMed were as follows: (Oligometastatic[All Fields] OR oligometastasis[All Fields]) AND ((“carcinoma, nonsmall-cell lung”[MeSH Terms] OR (“carcinoma”[All Fields] AND “nonsmall-cell”[All Fields] AND “lung”[All Fields]) OR “nonsmall-cell lung carcinoma”[All Fields] OR “NSCLC”[All Fields]) OR “nonsmall cell lung cancer”[All Fields]).

### 2.2. Inclusion and Exclusion Criteria

The studies met all of the following criteria: (1) subjects were NSCLC patients with oligometastasis; (2) the risk factors of overall survival were investigated; and (3) the study was published in English.

The studies should be excluded if (1) the included data were not fully enough for statistical analysis; (2) the study was reviews, letters, or comments; (3) the newest study or the study with the most complete information was included when the data were repeatedly duplicated or the same population data were applied for multiple researches; and (4) the studies had obvious logic errors.

### 2.3. Data Extraction

The following data were recorded in a predesigned form: the name of the first author, study area, year of publication, study time, sample size, and the characteristics of objects (such as age and sex). The data extraction was performed independently by two investigators. Differences were resolved by discussion with the third investigators to ensure consistency of evaluation.

### 2.4. Statistical Analysis

The R 3.12 (R Foundation for Statistical Computing, Beijing, China, Package: Meta) was used to perform this meta-analysis [18]. The I-squared and Cochran Q tests were used to evaluate the heterogeneity among included studies [19]. It was defined that significant heterogeneity occurred when *P* < 0.05 or *I*^2^ > 50%. If significant heterogeneity was observed among individual studies, the random effects model would be used to estimate the pooled effect of outcomes. If no obvious heterogeneity was observed, the fixed effect model would be used to pool the hazard radio (HR) as well as the corresponding 95% confidence interval (CI). The publication bias test was conducted using Egger's test [20]. If there is a publication bias, the shear compensation method would be used to evaluate the previous numerical and analytical results. Sensitivity analysis was analyzed by ignoring a single document at a time and seeing if this document can reverse the overall combined effect [21]. For all these analyzes, *P* < 0.05 indicated statistical significance.

## 3. Results

### 3.1. Characteristics of Included Studies

**Figure 1:**
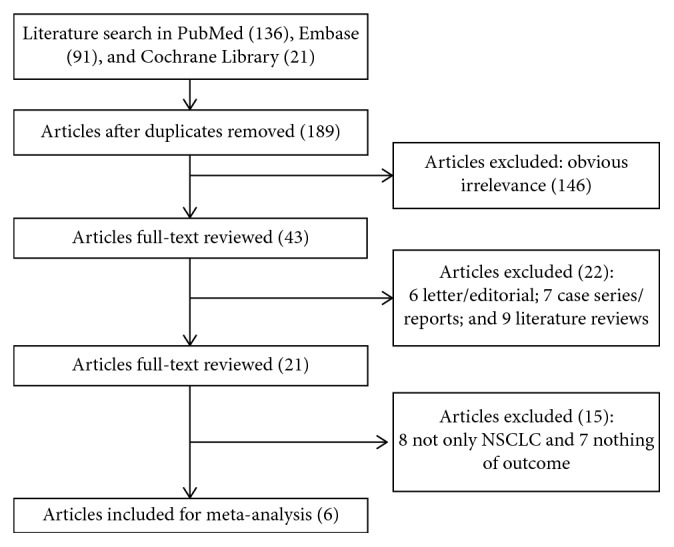
Flow diagram of the study selection process.

The flowchart of article selection is shown in Figure 1. The initial literature search identified 248 articles from PubMed (*n*=136), Embase (*n*=91), and Cochrane library (*n*=21). After excluding duplicates, 189 potentially relevant articles remained for further review. Then, 146 irrelevant studies were removed by scanning the titles or abstracts. Of the remaining 43 articles, 22 articles were excluded including 6 letter/editorial, 7 case series/reports, and 9 literature reviews. Then, 21 articles were full reviewed, 15 articles were excluded because the subjects in 8 articles were not only NSCLC, and outcomes in 7 articles were not shown. Finally, 6 studies [9, 15–17, 22, 23] were included and analyzed in this study.

The baseline characteristics of included studies are shown in Table 1. Briefly, a total of 6 studies including 565 oligometastatic NSCLC patients (female: *n*=232; male: *n*=333) were included in this meta-analysis. These patients received several different treatments strategy including surgical resection, whole brain radiation therapy, stereotactic radiosurgery, and chemoradiation. The publication year ranged from 2010 to 2016. The study time was between 1995 and 2015 (Table 1). The study region included China, Germany, United States, Japan, and Canada.

### 3.2. Meta-Analysis

In this study, the risk factors of overall survival of oligometastatic NSCLC patients were analyzed, including sex (female vs. male), stage (stage I-II vs. stage III), nodal status (N0/N1 vs. N2/N3), smoker (smoker vs. nonsmoker), age (>60 vs. ≤60), and histology (others vs. adeno).

Significant heterogeneity among studies was found in the analyses for UICC-stage (*I*^2^=57.3%, *P*=0.07) and histology (*I*^2^=70.7%, *P*=0.03), so the data involved in the meta-analysis were pooled based on the random effects model. Moreover, no significant heterogeneity was observed on data evaluating the association between oligometastatic NSCLC occurrence and age (*I*^2^=0%, *P*=0.64), sex (*I*^2^=35.5%, *P*=0.21), N-stage (*I*^2^=25%, *P*=0.25), and smoker (*I*^2^=0%, *P*=0.89), and the fixed effects model was used.

**Figure 2:**
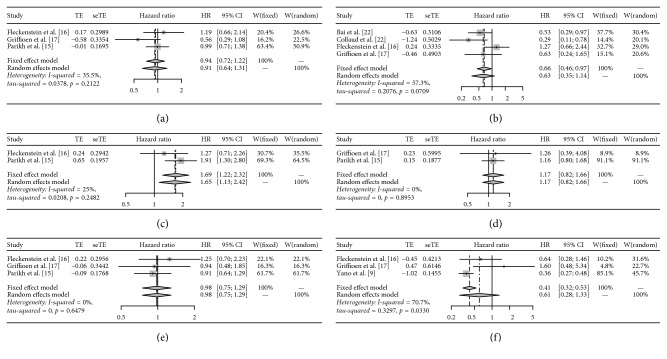
Meta-analysis for the association of survival of oligometastatic non-small-cell lung cancer patients with sex (a), stage (b), nodal status (c), smoke (d), age (e), and histology (f).

**Figure 3:**
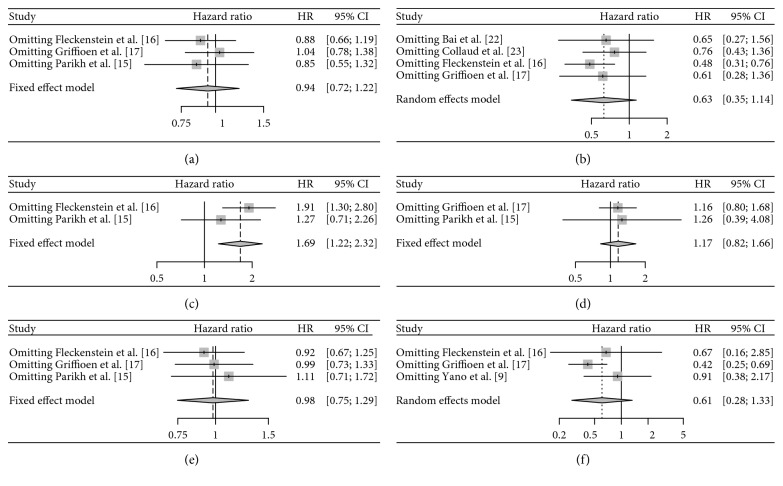
Sensitivity analysis for the association of survival of oligometastatic non-small-cell lung cancer patients with sex (a), stage (b), nodal status (c), smoke (d), age (e), and histology (f).

The main results are shown in Table 2. We found that nodal status was significantly related to the overall survival rate of NSCLC oligometastatic patients (HR: 1.69, 95% CI: 1.23–2.32, *Z*=3.20, *P*=0.001). However, only two articles were included. As for the relationship between overall survival rate of NSCLC oligometastatic patients and the indicators including sex, stage, smoker, age, and histology, no significant effect on the survival rate of patients was found (Figure 2). Notably, sensitivity analysis on data evaluating relationship between patients survival and the stage and histology showed that results could be reversed after we removed one of the documents (Figure 3). Egger's test did not detect publication bias in this meta-analysis (*P* > 0.05).

## 4. Discussion

Although advanced NSCLC is associated with low survival, oligometastatic NSCLC patients have a better prognosis [13]. In order to evaluate the overall survival for these patients more precisely, we performed the meta-analysis to evaluate the potential factors associated with the survival of oligometastatic NSCLC patients. We found that nodal status was significantly related with the overall survival rate of NSCLC oligometastatic patients. However, no significant relationship was found between sex, stage, smoker, age, and histology and the overall survival of oligometastatic NSCLC patients. However, some factors may affect the association of overall survival with sex, stage, smoker, age, and histology.

Cancer patients with 1–5 metastatic or recurrent lesions after treatment are considered to have oligometastases. The role of metastatic status in the overall survival of oligometastatic NSCLC is similar to that reported in a previous analysis that the metastases status, such as number and location of metastases, was associated with the survival of patients with NSCLC. Similarly, a meta-analysis also demonstrated that N1-stage or N2-stage (vs. N0) was a predictive factor for a decreased OS based on 757 patients [10]. For patients with synchronous oligometastases, Mordant et al. demonstrated that the absence of nodal mediastinal involvement was associated with improved overall survival [24]. In the present study, we also showed that nodal status was a positive prognostic factor. Thus, in order to pursue definitive treatment for these patients, it should be recommended that factors such as nodal involvement should be fully considered.

There were obvious histology differences between adenocarcinoma and nonadenocarcinoma NSCLC. These histology differences may result in different responses to the same treatment. Moreover, it is also reported that the histology can be used for predicting the survival of NSCLC patients with brain metastases [25, 26]. Notably, only two included studies reported association between N-stage and survival of oligometastatic NSCLC patients, and the results in these two studies were inconsistent [15, 16]. As for the evaluation of UICC-stage and overall survival of oligometastatic NSCLC patients, significant heterogeneity was found among studies. Furthermore, significant evidences were also found among the studies evaluating association between histology and overall survival of oligometastatic NSCLC patients [14]. In addition, no association of overall survival with age, sex, and smoke was found in oligometastatic NSCLC patients. However, a previous study showed that male sex, age ≥80 years, and smoking were associated with the shorter survival time in NSCLC patients aged ≥70 years [27]. Moreover, the association of sex, age, and smoke with metastases in NSCLC patients has been reported. The discrepancy between the findings in those studies and those in ours may be explained by confounding factors such as sex ratio, county distribution, and included sample size. Although we found that no significant relationship was found between sex, stage, smoker, age, and histology and the overall survival of oligometastatic NSCLC patients, further studies should be performed to explore these influence factors.

There were some limitations in this meta-analysis. Firstly, signiﬁcant heterogeneities occurred when we pooled data from individual studies evaluating the role of histology and stage in overall survival of oligometastatic NSCLC patients. For the meta-analysis, heterogeneity degree is an important indicator of meta-analysis validity [28]. According to the limited enrolled sample size, subgroup analysis could not be performed. It could not be repudiated that patients baseline characteristics, site of oligometastatic disease (e.g., brain, bone, and adrenal glands), and treatment strategy might be blamed for the sources of the obvious heterogeneities. Second, many factors, such as tumor size and metastasis, could not be analyzed in this meta-analysis due to lack of available data. The risk factors for progression-free survival should be determined in further studies. Thus, it should be recommended that further study with larger sample size and homogeneity of data would be designed to verify the current conclusion.

## 5. Conclusion

In conclusion, our study suggested that nodal status might be a prognostic factor for oligometastatic NSCLC patients. However, the number of included studies was small, and further attention should be paid to this research.

## Figures and Tables

**Table 1:** Characteristics of included studies.

Author	Published year	Study location	Study year	*N*	Surgical method	Median age, years (range)	Sex (male/female)	Smoking status^†^
Bai et al. [22]	2016	China	1995.9–2011.7	76	Brain surgery; SRS	58 (30–82)	52/24	47
Collaud et al. [23]	2012	Switzerland	2002–2008	29	S; WBRT; RT	62 (44–77)	20/9	NA
Fleckenstein et al. [16]	2016	Germany	2000.3–2015.4	75	Surgery; SBRT; SRS; RT	59.1 ± 8.4	43/32	NA
Griffioen et al. [17]	2013	Canada	1999.9–2.12.7	61	Surgery; RT; WBRT; CRT	61.7 ± 9.5	31/30	56
Parikh et al. [15]	2014	USA	2002–2012	186	SBRT; WBRT; SRS	NA	92/94	53
Yano et al. [9]	2010	Japan	2005–2009	138	Surgery; RT	67.8 (38–85)	95/43	NA

WBRT: whole brain radiation therapy; SRS: stereotactic radiosurgery; S: surgical resection; RT: radiotherapy; SBRT: stereotactic body radiotherapy; hSRT: hypofractionated stereotactic radiosurgery; CRT: chemoradiation; SABR: stereotactic ablative radiation therapy; ^†^current smoker.

**Table 2:** Fixed/random effects for oligometastatic non-small-cell lung cancer of overall survival.

Item	Group	Test of association			Model	Test of heterogeneity^†,‡^			Egger's test for publication bias^§^	
		HR (95% CI)	*Z*	*P*		*Q*	*P*	*I* ^2^ (%)	*t*	*P* value
Sex	Female vs. male	0.9373 [0.7194; 1.2211]	0.48	0.6312	Fixed	3.10	0.2122	35.5	0.4997	0.705
Stage	Stage I-II vs. stage III	0.6268 [0.3455; 1.1372]	−1.54	0.1243	Random	7.03	0.0709	57.3	0.7702	0.5217
Nodal status	N2/N3 vs. N0/N1	1.6853 [1.225; 2.3194]	3.20	0.0014	Fixed	1.33	0.2482	25.0	—	—
Smoker	Smoker vs. nonsmoker	1.1686 [0.8225; 1.6602]	0.87	0.3845	Fixed	0.02	0.8953	0	—	—
Age	>60 vs. 60	0.9812 [0.7474; 1.2881]	−0.14	0.8913	Fixed	0.87	0.6479	0	0.70602	0.6086
Histology	Others vs. adeno	0.6056 [0.2762; 1.3277]	−1.25	0.2105	Random	6.82	0.0330	70.7	4.7318	0.1326

^†^Random effects model was used when the *P* value for heterogeneity test is <0.05; otherwise, the fixed effect model was used. ^‡^*P* < 0.05 is considered statistically significant for Q statistics. ^§^Egger's test to evaluate publication bias, *P* < 0.05, is considered statistically significant. OR: odds ratio. CI: confidence interval.

## Data Availability

The data used to support the findings of this study are available from the corresponding author upon request.

## Abbreviations

NSCLC: Non-small-cell lung cancer
HR: Hazard radio
CI: Confidence interval.
